# Diabetic Ketoacidosis Severity at Diagnosis and Glycaemic Control in the First Year of Childhood Onset Type 1 Diabetes—A Longitudinal Cohort Study

**DOI:** 10.3390/ijerph15010026

**Published:** 2017-12-25

**Authors:** Amal R. Khanolkar, Rakesh Amin, David Taylor-Robinson, Russell M. Viner, Justin Warner, Evelien F. Gevers, Terence Stephenson

**Affiliations:** 1 GOS Institute of Child Health, University College London (UCL), 30 Guildford Street, London WC1 1EH, UK; Rakesh.Amin@gosh.nhs.uk (R.A.); r.viner@ucl.ac.uk (R.M.V.); terence.stephenson@gmc-uk.org (T.S.); 2 Institute of Environmental Medicine, Karolinska Institutet, 17177 Stockholm, Sweden; 3 Department of Public Health and Policy, University of Liverpool, London L69 3BX, UK; David.Taylor-Robinson@liverpool.ac.uk; 4 Department of Child Health, Children’s Hospital for Wales, Cardiff CF14 4XW, UK; Justin.Warner@wales.nhs.uk; 5 Centre for Endocrinology, William Harvey Research Institute, Queen Mary University London, London EV1M 6BQ, UK; Evelien.Gevers@bartshealth.nhs.uk; 6 Department of Paediatric Endocrinology, Barts Health NHS Trust—Royal London Children’s Hospital, London E1 1BB, UK

**Keywords:** type 1 diabetes, diabetic ketoacidosis, glycaemic control, ethnicity, inequalities, longitudinal analysis

## Abstract

It is unclear whether diabetic ketoacidosis (DKA) severity at diagnosis affects the natural history of type 1 diabetes (T1D). We analysed associations between DKA severity at diagnosis and glycaemic control during the first year post-diagnosis. We followed 341 children with T1D, <19 years (64% non-white) attending paediatric diabetes clinics in East London. Data were extracted from routine medical registers. Subjects were categorized with normal, mild, moderate, or severe DKA. Linear mixed-effects modelling was used to assess differences in longitudinal HbA1_c_ trajectories (glycaemic control) during 12 months post-diagnosis (1288 HbA1_c_ data-points) based on DKA, adjusting for sex, age, ethnicity, SES (Socioeconomic Status) and treatment type. Females (OR 1.6, 95% CI 1.1–2.4) and younger age, 0–6 vs. 13–18 years (OR 2.9, 95% CI 1.5–5.6) had increased risk for DKA at diagnosis. Moderate or severe DKA was associated with higher HbA1_c_ at diagnosis (adjusted estimates 8 mmol/mol, 2–14, and 10 mmol/mol, 4–15, respectively, compared to normal DKA). Differences in HbA1_c_ trajectories by DKA were no longer apparent at six months post-diagnosis. All subjects experienced a steep decrease in HbA1_c_ during the first three months followed by a gradual increase. While, DKA severity was not associated with glycaemic control at 12 months post-diagnosis, age at diagnosis, ethnicity, gender, and treatment type were significantly associated. For example, Black and mixed ethnicity children had increased risk for poor glycaemic control compared to White children (adjusted RRR 5.4, 95% CI 1.7–17.3 and RRR 2.5, 95% CI 1.2–6.0, respectively). DKA severity at diagnosis is associated with higher initial HbA1_c_ but not glycaemic control from six months post-diagnosis. Age at diagnosis, ethnicity, gender, and insulin pump are associated with glycaemic control at one year post-diagnosis.

## 1. Introduction

Children with type 1 diabetes (T1D) demonstrate considerable clinical variation at diagnosis despite the vast majority having lost most of their beta-cell function (60–80% of beta-cells stop producing insulin) [1,2]. Progressive reduction in endogenous insulin production results in metabolic derangements leading to life threatening hyperglycaemia, volume depletion, electrolyte imbalance, and formation of ketone bodies culminating in diabetic ketoacidosis (DKA). DKA severity at diagnosis varies considerably between patients and is affected by delayed diagnosis, younger age (<5 years), ethnic minority, and lower socioeconomic status (SES) [1,3]. Individual variability in DKA ranges from mild with minimal dehydration and acidosis to severe with excessive dehydration and acidosis [1].

Observational studies demonstrate long-term tracking of poor glycaemic control from diagnosis which is associated with increased risk for chronic micro- and macro-vascular complications [4,5,6,7,8,9]. Several factors at diagnosis are associated with poor glycaemic control during the first two years including ketoacidosis severity, younger age, gender, humoral immune responses, SES, ethnicity, and psychosocial factors such as parental involvement, psychosocial maturity, and family situation [2,10,11,12]. Studies on ketoacidosis severity at diagnosis and poorer glycaemic control are limited and report conflicting results. Additionally, the association between ketoacidosis severity and glycaemic control is confounded by age at diagnosis as younger children present with greater ketoacidosis severity but lower glycated haemoglobin (HbA1_c_) [2,13].

We investigated longitudinal relationships between ketoacidosis severity at diagnosis and glycaemic control during the first year post-diagnosis in a multi-ethnic cohort of subjects attending pediatric diabetes clinics in East London. We also investigated factors associated with ketoacidosis at diagnosis and glycaemic control at 12 months post-diagnosis. We aimed to identify subjects with a “high risk” profile; presenting with greater ketoacidosis at diagnosis and poor early glycaemic control. This may enable developing targeted interventions for improving glycaemic control in such patients earlier on and is important as poor early glycaemic control tracks into young adulthood.

## 2. Patients and Methods

### 2.1. Design, Setting, and Data Source

We designed a longitudinal cohort study of newly diagnosed patients with T1D using data from three paediatric diabetes clinics that are part of the same Healthcare Trust (Barts Health NHS Trust) located in East London, UK [14]. The clinics largely capture patients living in surrounding areas of East London, where ~56% of the local population belongs to an ethnic minority, with around 50% of South Asian origin (primarily Bangladeshi) and 40% of Black origin (primarily Somali) [15].

The study was restricted to children <19 years of age who received a diagnosis of T1D between 1 January 2005 and 31 December 2015 and attended one of the three clinics during this period. Clinical and sociodemographic data were collected prospectively, both at the time of diagnosis and during routine clinic visits. As recommended by the National Institute of Health and Care Excellence (NICE), a child with T1D is offered an integrated package of care by a multidisciplinary team at a paediatric diabetes clinic four times/year. The team consists of paediatric endocrinologists/diabetologists, diabetes specialist nurses, dieticians, psychologists, interpreters, and a database manager. HbA1_c_ levels are recorded at each visit. All demographic and clinical parameters are systematically measured and electronically documented across the clinics enabling comparison. A total of 598 children were diagnosed with T1D during the study period, of whom 580 (97%) had complete data on sex, age at diagnosis, duration of diabetes, and ethnicity and were eligible to be included in the analysis.

### 2.2. Primary Outcome, Exposures, and Covariates

The primary outcome was glycaemic control measured by glycated haemoglobin or HbA1_c_ levels. HbA1_c_ was measured at each visit using the point of care Siemens/Bayer DCA 2000+ Analyzer. HbA1_c_ values recorded as percentages were converted to mmol/mol (formula: HbA1_c_ value in %−2.15) × 10.929). Subjects were classified as having good (HbA1_c_ <58 mmol/mol), moderate (HbA1_c_ 58–79 mmol/mol) and poor (HbA1_c_ ≥80 mmol/mol) glycaemic control.

The primary exposure was DKA severity at diagnosis measured by pH levels and the pH value (blood capillary samples) measured closest to initial presentation was used. Based on the ISPAD (International Society for Pediatric and Adolescent Diabetes) Clinical Practise Consensus Guidelines, subjects were grouped into normal (pH ≥ 7.3), mild (pH 7.2–7.29), moderate (pH 7.1–7.19), and severe (pH < 7.1) DKA [1]. These are also the national guidelines for the definition of DKA in the UK as recommended by NICE [16].

Covariates included age at diagnosis, sex, ethnicity, SES, treatment type, and clinic attended. Participants (or their parents) self-identified their ethnicity (choosing 1 of 15 categories or the option to decline identifying their ethnicity) during clinic visits. We used the first recorded entries for ethnicity at the time of diagnosis. The 15 ethnic categories were collapsed into four broad groups: White, mixed-ethnicity (any mixed ethnicity combination), Black, and Asian (any Asian origin) reflecting the ethnic distribution of the study area in East London. The latter group included subjects mostly of Bangladeshi, Pakistani, and some Indian origin and a small proportion originating from other Asian countries.

SES was derived from postcode of residence using indices of multiple deprivation (IMD) 2010 for England [17]. The IMD is a small geographical area measure of deprivation with scores derived from a weighted combination of indicators across seven measures of deprivation including income, employment, education skills and training, health, barriers to housing and services, living environment, and crime. IMD scores are calculated at the level of lower-layer super output areas, with each area comprising 1500 individuals on average. Lower IMD rank scores indicate higher levels of deprivation.

Age at diagnosis was calculated by subtracting date of diagnosis from date of birth. Duration of diabetes was calculated in months by subtracting the date at first visit in the audit year from the date of diagnosis of T1D. For some analysis, age at diagnosis was categorised into 0–6, 7–12, and 13–18 years. Treatment type was a dichotomous variable with subjects categorised into insulin pump or non-insulin pump therapy.

### 2.3. Statistical Analysis

Baseline characteristics were compared across all DKA groups. Categorical variables were compared as frequencies using Chi2 or Fisher’s Exact test (if numbers ≤5). Mean differences in baseline continuous variables by DKA were analysed using simple linear regression. We tested for a trend in continuous variables (age at diagnosis, pH levels, and HbA1_c_) across the different DKA groups using the Stata command “*nptrend*” (which uses an extension of the Wilcoxon rank-sum test for trend across ordered groups).

We used multivariable logistic regression to analyse factors (categorical age at diagnosis, sex, ethnicity, and SES (quartiles)) associated with DKA at diagnosis (defined as pH < 7.3).

Linear mixed effects modelling (growth curve analysis) was used to assess whether HbA1_c_ trajectories differed by DKA at diagnosis over the first 12 months post-diagnosis. This enables comparison of population average HbA1_c_ levels and change over time for the three DKA categories while controlling for covariates. We approximated time trends using a cubic model for time since diagnosis as this provided a better statistical fit compared to linear and quadratic models. We first fit a random intercept model (i.e., an unconditional means model) to define the intraclass correlation coefficient (ICC). This was compared to a random intercept and random slope model which had a better fit and was used in subsequent models. DKA at diagnosis (categorical), ethnicity, age at diagnosis (continuous), sex, SES (continuous), and diabetes clinic were entered as time-invariant predictors whereas treatment regimen was entered as time-variant. We ran four models: Model 1: Random intercept only (unconditional model); Model 2: unadjusted growth model with a cubic function of time since diagnosis (disease duration) as the time metameter; Model 3: adjusted for our primary predictor; DKA at diagnosis and additionally an interaction term between DKA and duration (to assess whether DKA status interacts with duration on mean HbA1_c_ trajectories) and Model 4: additionally adjusted for sex, age at diagnosis, ethnicity, treatment type, SES, and diabetes clinic attended. The model with an interaction between DKA status and linear duration only was chosen as it had a better fit than the model including interactions with higher order polynomials (quadratic and cubic duration). Additionally, both interaction models had a very similar visual fit. Model parameters were estimated by maximum likelihood and a heterogeneous autoregressive covariance structure was used in all models. We used generalized likelihood ratio statistics, −2 log-likelihood (−2 LL), Aikake information criterion (AIC), and sample-adjusted Bayesian information criterion (BIC) to compare model fit between subsequent nested models, and Wald statistics to test hypotheses about model parameters. We plotted cubic growth curves at the group level (i.e., DKA status at diagnosis) from the model with linear interaction terms to visualise model fit.

### 2.4. Factors Associated with Glycaemic (HbA1_c_) 12 Months Post-Diagnosis

We calculated relative risk ratios (RRR) of moderate (HbA1_c_ 58–79 mmol/mol) and poor (HbA1_c_ ≥80 mmol/mol) glycaemic control with good control as the baseline, comparing those with DKA to no DKA at diagnosis using multivariable multinomial logistic regression. We ran two models: Model 1—minimally adjusted for DKA at diagnosis only; and Model 2—additionally adjusted for sex, age at diagnosis, ethnicity, treatment type, and paediatric diabetes clinic attended.

### 2.5. Sensitivity Analysis

We compared the study sample of 364 children with data on pH at diagnosis with those missing data on pH at diagnosis (N = 126) to ensure that results were generalizable to the eligible population (N = 580, of whom 490 had pH data).

All analyses were run in Stata 13 (StataCorp LLC, Lakeway Drive, College Station, TX, USA).

For this study, all participants were anonymized making them unidentifiable. The study is registered with the R and D Office, GOS Institute of Child Health, UCL (University College London) (Project number 14PP08).

## 3. Results

Of the 580 children with data on various covariates, 490 children had at least one recorded HbA1_c_ value within the first 12 months post-diagnosis. Of these, 126 children were missing data on pH at diagnosis, leaving a final sample of 364 with data on all other covariates for inclusion in analysis. There were no significant differences in age and HbA1_c_ at diagnosis between the study sample (N = 364) and those excluded because of missing data on pH (N = 126). We observed no significant differences in the distribution of cases by gender, ethnicity, and clinic attended between the study sample and those excluded because of missing data (Supplemental Table S1).

Mean age at diagnosis was 8.8 years. 41% presented with any form of DKA (pH ≤ 7.2) at diagnosis. Females and younger children were more likely to present with lower pH levels at diagnosis. For example, mean age at diagnosis was two years lower in the severe compared to the normal DKA groups (7.5 vs. 9.6 years, *p* < 0.001, Table 1). The severe, moderate and mild DKA groups had higher HbA1_c_ levels at diagnosis compared to the normal group, but differences were not statistically significant. However, a test for trend of HbA1c levels across the DKA categories was statistically significant (*p* = 0.03, Table 1). There were no differences in proportions between the three DKA groups by ethnicity, SES, or diabetes clinic attended.

### 3.1. Factors Associated with DKA at diagnosis

Females and those younger at diagnosis had increased odds to present with DKA (pH < 7.3). Compared to males, females had a 60% increased risk to present with DKA. Those aged 0–6 and 7–12 years at diagnosis had significantly increased odds to present with DKA (OR 2.9, 95% CI 1.5–5.6 and OR 2.3, 95% CI 1.2–4.3 respectively, Table 2) compared to those aged 13–18 years. Ethnicity and SES were not associated with risk of DKA at diagnosis.

### 3.2. Longitudinal Analysis of HbA1c Trajectories by DKA Status (pH Level) at Diagnosis

**Figure 1:**
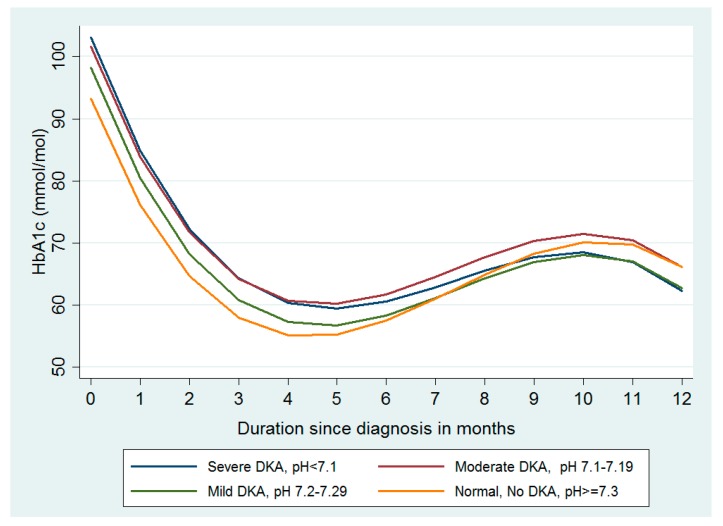
Linear adjusted and predicted HbA1_c_ trajectories based on DKA status at diagnosis in 364 children and young people diagnosed with type 1 diabetes and attending paediatric diabetes clinics in East London. DKA: diabetic ketoacidosis.

Mean HbA1_c_ for the study sample at diagnosis was 96 mmol/mol. On average, subjects experienced a sharp decline in HbA1_c_ in the first two months following diagnosis (−21 mmol/mol, 95% CI −22, −19, Model 2, Table 3). The initial decline in HbA1_c_ (negative linear term, −21 mmol/mol), was followed by a slight gradual increase in HbA1_c_ (positive quadratic term, 3 mmol/mol) before a gradual decrease and stabilisation of HbA1_c_ (small negative cubic term, −0.1 mmol/mol, Table 3 and Figure 1). Subjects in the moderate and severe DKA groups had higher HbA1_c_ at diagnosis (8 mmol/mol and 9 mmol/mol respectively, Model 3, Table 3). Adjustment for covariates did not significantly affect the estimates for DKA status at diagnosis (moderate DKA; 8, 95% CI 2–14 and severe DKA; 10 mmol/mol, 4–15, Model 4, Table 3).

The test for interaction between DKA at diagnosis and duration on mean HbA1_c_ trajectories was statistically significant (*p* = 0.01), indicating initial differences in HbA1_c_ trajectories by DKA was no longer apparent after the initial steep decrease in HbA1_c_ (linear term for duration). The moderate and severe DKA groups experienced a small additional decrease in HbA1_c_ (−1 mmol/mol, 95% CI −2, −0.1 and −1 mmol/mol, −2, −1 respectively) when compared with the normal DKA group during the first three months. Figure 1 shows the adjusted predicted population average HbA1_c_ trajectories by DKA group over 12 months post-diagnosis. Differences in HbA1_c_ levels by DKA at diagnosis are no longer evident from around six months post-diagnosis. Additionally, differences in HbA1_c_ trajectories by severe and moderate DKA were negligible (i.e., trajectories run concurrently).

Ethnic minority groups had significantly higher HbA1_c_ at diagnosis. The model with all covariates, including the interaction term between DKA status and duration, had the best model fit (Model 4, Table 3, i.e., the lowest AIC = 10694.27 and −2 LL = 10648.272).

### 3.3. Factors Associated with Glycaemic Control (HbA1_c_) 12 Months Post-Diagnosis

DKA at diagnosis was not associated with moderate or poor glycaemic control 12 months post-diagnosis. Factors like age at diagnosis, ethnicity, gender, clinic and treatment type were associated with glycaemic control. Children diagnosed between 0–6 years of age were at increased risk for moderate glycaemic control (RRR (Relative risk ratio) 2.3, 95% CI 1.1–4.9, Table 4). Additionally, Black and mixed ethnicity children were at increased risk for poor glycaemic control compared to White children (adjusted RRR 5.4, 95% CI 1.7–17.3 and RRR 2.5, 95% CI 1.2–6.0, respectively, Table 4). Males were at decreased risk for poor glycaemic control.

## 4. Discussion

In this multi-ethnic cohort, we found females and those diagnosed at younger ages had increased risk for moderate and severe DKA at initial presentation. Subjects with moderate or severe DKA presented with higher HbA1_c_ levels. However, differences in early glycaemic control by DKA status were no longer evident from six months post-diagnosis. Most subjects experienced a steep decline in HbA1_c_—the “honeymoon phase”—during the first two months post-diagnosis irrespective of their initial DKA status. Lastly, in line with longitudinal modelling, initial DKA status was not associated with glycaemic control one year post-diagnosis, but ethnicity, age, and gender were relevant factors.

Mixed effect modelling allows for the inclusion of a large number of data points even when the data is ‘unbalanced’ (subjects with differing number of data points measured at different times). To our knowledge, this is the first study in the UK to investigate associations between pH/DKA at diagnosis and early glycaemic control using longitudinal modelling as data on pH is not routinely collected in national datasets. We had near complete data on covariates (sex, age at diagnosis, duration, and ethnicity). Ethnicity was self-identified which is considered to be the ‘gold standard’ in studies on ethnicity and health. Data was collected from routine electronic medical records limiting recall and selection bias.

Limitations include that the study sample was drawn from three diabetes clinics (operating together as a network since 2012) and results may not be generalizable to the rest of the country. One cannot exclude the possibility of residual confounding due to other factors impacting glycaemic control at diagnosis and during follow-up which may interact with ketoacidosis severity including delayed diagnosis and sociodemographic factors (family structure and family history of diabetes). As data on insulin doses is not collected, we were unable to investigate potential differences in rates of transient remission by DKA and ethnicity. Contrary to expectations, SES was not associated with risk for DKA at diagnosis or with glycaemic control in the longitudinal analysis. A significant proportion of the study sample was deprived (reflecting the neighbourhoods from which the sample was drawn) leading to low variability in SES. In a previous study on the same cohort, we found no associations between SES and pH levels, HbA1_c_ at diagnosis or during follow-up [14]. A significant proportion of our subjects were missing data on pH levels. However, we found no significant differences between those excluded because of missing data from those included indicating generalizability of the results to the entire study population. Subjects diagnosed abroad or in other clinics in the country were expected to have missing pH data as their initial medical records were inaccessible.

Our study corroborates associations between younger age at diagnosis and increased risk for DKA at presentation [2,3,18,19,20]. Additionally, we found that females have an increased risk for DKA at diagnosis. However, previous studies report inconsistent associations between gender and risk for DKA at diagnosis and we can offer no firm hypothesis to explain our observation [3,19]. Åkesson et al. hypothesise that non-recognition of symptoms in females leading to a later diagnosis and hence greater disease severity at initial presentation could be a potential explanation [19]. However, we found no statistical differences in age at diagnosis between males and females. During adolescence, females are more insulin resistant than males, which may impact on frequency and severity of DKA. However there was no gender difference between the ages of 13 and 18 years so this is unlikely to be the explanation. The severity of the autoimmune process and the level of endogenous insulin production after diagnosis of T1D does not differ between the sexes so this is also unlikely to explain the observed gender difference [21].

Our cohort is unique with its high proportion of ethnic minorities and social deprivation, so within this there may be relevant cultural and social factors that impact on the timing of seeking medical assistance for girls compared with boys but we have no evidence to support this hypothesis.

The proportion of subjects presenting with DKA (41%) is higher than that reported elsewhere but differences are likely due to the differing cut-offs for DKA [3]. We found no ethnic differences in DKA severity at diagnosis as reported in a previous UK-based study [22]. However, more recent studies have not found ethnic differences in prevalence of DKA at diagnosis [3]. High HbA1_c_ levels at diagnosis, followed by the steep decline during the first two months and the subsequent gradual increase observed here is similar to that reported elsewhere [23,24,25]. Additionally, ethnic differences in initial and 12 months HbA1_c_ levels was previously reported in this cohort and other studies [23].

Few studies have analysed associations between DKA/pH at presentation and glycaemic control during the first year post-diagnosis limiting comparisons. Åkesson et al. reported a significant inverse association between pH level and HbA1_c_ at diagnosis in a Swedish cohort but did not report long-term associations [19]. In a single centre study on Scottish children, Lawes et al. found male patients with DKA at diagnosis had higher HbA1_c_ six months post-diagnosis, but the study did not report longitudinal HbA1_c_ trajectories based on DKA at diagnosis [4].

The evidence for a metabolic memory in T1D (the association between early tight glycaemic control due to intensive treatment and reduced risk for long term micro- and macro-vascular complications risk and mortality) has highlighted the need to achieve good glycaemic control in the months following diagnosis [26]. Identifying modifiable predictors of this early glycaemic control would potentially aid this clinical approach. In addition to socioeconomic and demographic factors (access to health care and awareness in primary care communities), the severity of DKA at presentation with T1D may partly be a reflection of endogenous insulin production. For example, children <5 years are more likely to present in severe DKA and are thought to have a more aggressive autoimmune mediated destruction of pancreatic beta cells and therefore, potentially reduced endogenous insulin. This is relevant as the latter is an important predictor of long-term glycaemic control and risk of complications and is also a target for T1D intervention studies. For those under 5 years of age, our data supports a link between DKA severity and lower endogenous insulin production (and consequently a shorter “honeymoon period”) as this age group has increased risk to present with severe DKA and have higher HbA1_c_ at 12 months post-diagnosis.

## 5. Novelty Statement

Moderate and severe DKA at diagnosis is associated with poorer initial glycaemic control during the first few months post-diagnosis in a multi-ethnic cohort of children with newly diagnosed type 1 diabetes.

DKA severity at diagnosis no longer determines HbA1_c_ trajectories from around six months post-diagnosis.

Age at diagnosis, gender, ethnicity, and insulin pump use are determining factors for glycaemic control one year after diagnosis.

Greater focus on education and awareness of T1D in the community, early diagnosis in primary care, and intensive treatment will improve favourable glycaemic control, especially in younger subjects.

## 6. Conclusions

This study confirms previous findings that females and younger age is associated with increased risk for moderate and severe DKA at T1D diagnosis. A novel finding is that moderate and severe DKA at diagnosis is associated with poorer initial glycaemic control during the first few months after first clinical presentation. However, the differing HbA1_c_ trajectories by DKA severity are no longer apparent from six months post-diagnosis. Age, ethnicity, and insulin pump-use appear to be strong factors determining glycaemic control one year after diagnosis. A greater focus on education and awareness of T1D in the community, early diagnosis in primary care, and intensive insulin therapy in order to achieve favourable glycaemic control, especially in younger subjects, will reduce the risk for short- and long-term outcomes associated with T1D.

## Figures and Tables

**Table 1:** Characteristics of 364 children and young people diagnosed with type 1 diabetes between 2005 and 2015 and attending paediatric diabetes clinics in East London. Values are means (SD) or proportions.

Covariate	Diabetic Ketoacidosis at Diagnosis				Statistical Significance		
	Normal (pH ≥ 7.3)	Mild (pH 7.2–7.29)	Moderate (pH 7.1–7.19)	Severe (pH < 7.1)	All	*p*-Value ^d^	*p*-Value ^e^
**N**	214	53	42	55	364		
**Age at diagnosis, continuous (Years)**	9.6 (4.1)	7.9 (4.2)	7.5 (4.2)	7.5 (4.2)	8.8 (4.2)	<0.001	<0.001
**Age at diagnosis, categorical**							
0–6 years	32	42	38	42	35	<0.05	NA
7–12 years	44	43	52	51	46		
13–18 years	24	15	10	7	18		
**Sex (Females)**	47	49	69	60	52	<0.05	NA
**pH**	7.38 (0.05)	7.25 (0.03)	7.15 (0.03)	6.97 (0.09)	7.27 (0.16)	<0.001	<0.001
**HbA1_c_ at diagnosis ^a^ (mmol/mol)**	87 (25.3)	91 (21.2)	93 (19.7)	94 (21.3)	89 (23.6)	0.32	0.03
**HbA1_c_ at 12 months ^b^ (mmol/mol)**	68 (19.2)	69 (17.7)	69 (16.9)	66 (12.8)	68 (17.4)	0.84	0.84
**Ethnicity**							
White	35	30	38	44	36	0.63	NA
Mixed-ethnicity	8	15	14	7	10		
Black	23	23	29	18	23		
Asian-Other	34	32	19	31	31		
Paediatric diabetes Clinic							
1	29	25	14	24	26	0.42	NA
2	24	32	33	22	26		
3	47	43	52	55	48		
**Treatment ^c^**							
Insulin pump therapy (%)	12	12	6	14	12	0.73	NA
**Number of HbA1c measurements**	2.5 (1.4)	2.9 (1.8)	2.5 (1.3)	2.6 (1.4)	2.6 (1.5)	0.06	0.45
**Socioeconomic Status (SES) (median)**	7367	7481	6474	7767	7367	0.67	NA
**Proportion in most deprived SES quartile**	25	27	22	35	27	0.14	NA

^a^ 227 initial HbA1_c_ values measured during the first month post-diagnosis, ^b^ 191 HbA1_c_ values measured between the 10th and 12th months post-diagnosis, ^c^ Proportion of subjects on insulin pump therapy 12 months after diagnosis, ^d^ *p* values are for tests of equal means or proportion, ^e^ *p* values are for tests of trends for continuous outcomes by categorical DKA (diabetic ketoacidosis) only. NA = Not Applicable. Bold heading: variable name.

**Table 2:** Factors associated with diabetic ketoacidosis (pH < 7.3) at diagnosis of type 1 diabetes in 364 children and young people attending paediatric diabetes clinics in East London.

Covariate	OR (95% CI)
Sex	
Male	Reference
**Female**	**1.6 (1.1–2.4)**
Age at diagnosis	
**0–6 years**	**2.9 (1.5–5.6)**
**7–12 years**	**2.3 (1.2–4.3)**
13–18 years	Reference
Ethnicity	
White	Reference
Mixed-ethnicity	1.1 (0.5–2.3)
Black	0.8 (0.4–1.4)
Asian	0.8 (0.5–1.3)
SES	
Quartile 1 (poorest)	Reference
Quartile 2	0.8 (0.5–1.5)
Quartile 3	1.2 (0.7–2.1)
Quartile 4 (richest)	0.6 (0.3–1.2)

Text in bold indicates statistical significance at *p* < 0.05, socioeconomic status.

**Table 3:** Mixed effects models for longitudinal change in glycaemic control (HbA1_c_) during the first year post-diagnosis of type 1 diabetes in 364 children attending paediatric diabetes clinics in East London.

Covariates	Model 1: Random Intercept Only	Model 2: Unconditional Growth Model Plus Random Slope	Model 3: Plus DKA at Diagnosis and Interactions between DKA and Duration	Model 4: Plus All Other Covariates *
*Fixed effects*	Mean difference in HbA1_c_, mmol/mol (95% CI)	Mean difference in HbA1_c_, mmol/mol (95% CI)	Mean difference in HbA1_c_, mmol/mol (95% CI)	Mean difference in HbA1_c_, mmol/mol (95% CI)
Constant/intercept (HbA1_c_, mmol/mol)	96 (93, 98)	96 (94, 99)	93 (90, 96)	87 (80, 94)
*Slope coefficients*				
Duration in months				
Linear	−20 (−21, −18)	−21 (−22, −19)	−20 (−22, −19)	−20 (−22, −19)
Quadratic	3 (3, 3)	3 (3, 4)	3 (3, 4)	3 (3, 4)
Cubic	−0.1 (−0.2, −0.1)	−0.1 (−0.2, −0.1)	−0.1 (−0.2, −0.1)	−0.1 (−0.2, −0.1)
DKA at diagnosis				
Normal (no DKA)			Reference	
Mild			6 (0, 11)	5 (−1, 10)
Moderate			8 (2, 14)	8 (2, 14)
Severe			9 (3, 15)	10 (4, 15)
Sex				
Male				Reference
Female				−1 (−3, 2)
Age at diagnosis (years)				0.2 (−1, 1)
Ethnicity				
White				Reference
Mixed-ethnicity				7 (2, 12)
Black				5 (1, 9)
Asian				6 (3, 10)
Treatment type				
Non-insulin pump				Reference
Insulin pump				−7 (−11, −2)
Paediatric Diabetes Clinic				
Clinic 1				Reference
Clinic 2				−0.1 (−4, 3)
Clinic 3				5 (1, 8)
Socioeconomic Status				0.1 (−0.1, 1)
*Interaction between DKA and duration*				
Linear * DKA Normal			Reference	Reference
Linear * DKA Mild			−1 (−2, 1)	−1 (−1, 1)
Linear * DKA Moderate			−1 (−2, −0.1)	−1 (−2, −0.1)
Linear * DKA Severe			−1 (−2, −0.4)	−1 (−2, −1)
Interclass Correlation (ICC)	0.41	0.60	0.58	0.53
*Model fit*				
Aikake information criterion (AIC)	10,790.12	10,725.24	10,721.18	10,694.27
Bayesian information criterion (BIC)	10,821.04	10,766.46	10,793.32	10,812.79
−2 LL	10,778	10,709.22	10,693.182	10,648.272
Likelihood ratio test for model comparison (*p*-value)	NA	<0.0001	<0.01	<0.0001

* Model 4 adjusted for sex, age at diagnosis, ethnicity, SES, treatment type (insulin pump vs. no insulin pump) and pediatric diabetes clinic attended.

**Table 4:** Relative risk ratios (RRR) associated with moderate (HbA1_c_ 58–79 mmol/mol) and poor glycaemic control (HbA1_c_ ≥80 mmol/mol) 12 months post-diagnosis of type 1 diabetes in 396 children and young people attending paediatric diabetes clinics in East London.

Glycaemic Control	Model 1, Minimally Adjusted RRR	95% CI	Model 2, Adjusted for All Covariates RRR	95% CI
***Good***	1.0		1.0	
***Moderate***				
**DKA**				
No (pH ≥ 7.3)	1.0		1.0	
Yes (pH < 7.3)	1.0	0.6–1.6	0.8	0.5–1.4
**Sex**				
Females			1.0	
Males			0.7	0.4–1.1
**Age at diagnosis**				
** 0–6 years**			**2.3**	**1.1–4.9**
7–12 years			1.7	0.8–3.5
13–18 years			1.0	
**Ethnicity**				
White			1.0	
Mixed-ethnicity			2.3	0.9–5.6
Black			1.0	0.5–1.8
Asian-Other			0.7	0.4–1.3
**Treatment type**				
No insulin pump			1.0	
**Insulin pump**			**0.4**	**0.2–0.8**
**Paediatric Diabetes Clinic**				
1			1.0	
2			1.2	0.6–2.4
3			0.8	0.4–1.4
***Poor***				
**DKA**				
No (pH ≥ 7.3)	1.0		1.0	
Yes (pH < 7.3)	1.0	0.6–1.8	1.1	0.6–2.1
**Sex**				
Females			1.0	
**Males**			**0.5**	**0.2–0.9**
**Age at diagnosis**				
0–6 years			0.3	0.1–0.7
7–12 years			0.7	0.3–1.6
13–18 years			1.0	
**Ethnicity**				
White			1.0	
**Mixed-ethnicity**			**5.4**	**1.7–17.3**
**Black**			**2.5**	**1.2–6.0**
Asian-Other			1.7	0.7–3.9
**Treatment type**				
No insulin pump			1.0	
**Insulin pump**			**0.3**	**0.1–0.9**
**Paediatric Clinic**				
1			1.0	
**2**			**3.7**	**1.5–9.0**
3			1.5	0.6–3.7

Text in bold indicates statistical significance at *p* < 0.05. RRR: relative risk ratio.

## Supplementary Materials

The following are available online at www.mdpi.com/1660-4601/15/1/26/s1, Table S1: Comparison of characteristics between the study population included in the analysis (N = 364) and those excluded because of missing data on pH (DKA) at diagnosis (N = 126).
