# Supplementary material for: Diabetic Ketoacidosis Severity at Diagnosis and Glycaemic Control in the First Year of Childhood Onset Type 1 Diabetes—A Longitudinal Cohort Study
Source: Int J Environ Res Public Health. 2017 Dec 25;15(1):26. doi: 10.3390/ijerph15010026 (PMC5800126; doi:10.3390/ijerph15010026)
Supplement: Supplementary file 1 [file ijerph-15-00026-s001.pdf]

Supplemental

**Table S1.** Comparison of characteristics between the study population included in the analysis (N = 364) and those excluded because of missing data on pH (DKA:) at diagnosis (N = 126).

| Characteristic | Study Population included in Analysis (N = 364) | Subjects excluded (N = 126) | *p*-value * |
|---|---|---|---|
| Age at diagnosis (years) | 8.8 | 9.1 | 0.5 |
| HbA1c (mmol/mol) | 68.5 | 68.5 | 0.9 |
| Ethnicity (%) | | | |
| White | 36 | 39 | 0.4 |
| Mixed-ethnicity | 10 | 6 | - |
| Black | 23 | 18 | - |
| Asian | 32 | 36 | - |
| Sex | | | - |
| Males | 48 | 55 | 0.2 |
| Females | 52 | 45 | - |
| Clinic attended | | | - |
| 1 | 26 | 28 | 0.7 |
| 2 | 26 | 22 | - |
| 3 | 48 | 50 | - |

* *p* values are for a test of equal means or proportions. DKA: diabetic ketoacidosis.
